# Conclusion

**DOI:** 10.1111/ene.16474

**Published:** 2024-09-30

**Authors:** Paul Boon, Elena Moro

**Affiliations:** ^1^ European Academy of Neurology University of Ghent Ghent Belgium; ^2^ European Academy of Neurology University of Grenoble Grenoble France

We hope that the readers of this *European Journal of Neurology* Special Issue have enjoyed the lively descriptions of the amazing journey in the past 10 years that our discipline, neurology, has achieved thanks to the extraordinary contribution of neurologists, neurological associations, and people with neurological diseases. In this context, we are also particularly proud to highlight the remarkable developments and achievements of the European Academy of Neurology (EAN), which celebrates its 10‐year anniversary in 2024. The experience gained within the former European Federation of Neurological Societies (EFNS) and the European Neurological Society (ENS) has allowed the EAN to immediately start fulfilling its main mission and vision – to reduce the burden of neurological disorders and to provide a home of neurology – with many successful initiatives. The collegial and hard work of the members of the consecutive EAN Boards, the Head Office, the Scientific Panels, and the Committees has contributed to consolidate a society that now includes and represents 48 vibrant National Neurological Societies in Europe. EAN has exponentially grown within these 10 years in terms of increasing the number of members, particularly young neurologists, growing the number and quality of scientific and educational activities, increasing the number and strengthening of national and international partnerships, increasing overall quality standards, and increasing involvement at the European Union (EU) level in Brussels. During the COVID‐19 pandemic, EAN has continued to help and serve the neurological community with several initiatives, including setting up a large European patient registry. In the past 10 years, the EAN Annual Congress has become one of the best attended and impactful neurological meetings for general neurologists and subspecialty neurologists alike. EAN is increasingly recognized as the European general neurology organization with a global reach. EAN is now at the forefront of global brain awareness and advocacy efforts.

The EAN has published a comprehensive Neurological Research Agenda for Europe, a patient‐centered research strategy that was particularly missing for clinical neurology. EAN has also started a Brain Health Strategy, the Brain Health Mission (BHM), and a Strategic Brain Health Road Map involving patient representatives and patient organizations, the EAN Scientific Panels, the National Neurological Societies, a number of closely affiliated scientific societies, and a large number of other stakeholders and EU partners.

There are several other leading EAN actions and proposals in the pipeline. In these difficult, belligerent and divergent times, EAN wants to recognize diversity, inclusion, and equity as essential determinants when dealing with neurological diseases. To adequately address the exponential growth in knowledge and technology development, and the constant and rapid changes in environment and climate, and geopolitical conditions, EAN will continue to work to prepare the young generations of neurologists for the neurology of tomorrow. EAN will continue to expand its relationship with other national, international, and global neurological associations, European stakeholders, and patient organizations to foster common initiatives.

There is no doubt that the coming years will continue to be a very exciting time for EAN: there is so much to be continued, to be developed, and to be discovered! Let's do all this together!

## AUTHOR CONTRIBUTIONS

**Paul Boon:** Conceptualization; writing – original draft; writing – review and editing. **Elena Moro:** Conceptualization; writing – original draft; writing – review and editing.

## CONFLICT OF INTEREST STATEMENT

The authors declare no conflict of interest.

## Data Availability

Not applicable.

